# Liver-brain axis with alcohol: Role of fibroblast growth factor 21 (FGF21)

**DOI:** 10.1016/j.gendis.2023.04.003

**Published:** 2023-05-24

**Authors:** Ke Zhang, Elena Blokhina, Evgeny Krupitsky, Marina Vetrova, Ti-Fei Yuan, Hua Wang

**Affiliations:** Shanghai Key Laboratory of Psychotic Disorders, Brain Health Institute, National Center for Mental Disorders, Shanghai Mental Health Center, Shanghai Jiao Tong University School of Medicine, Shanghai 200030, China; Pavlov University, Valdman Institute of Pharmacology, St. Petersburg 197022, Russia; Bekhterev National Medical Research Center for Psychiatry and Neurology, St. Petersburg 192019, Russia; Shanghai Key Laboratory of Psychotic Disorders, Brain Health Institute, National Center for Mental Disorders, Shanghai Mental Health Center, Shanghai Jiao Tong University School of Medicine, Shanghai 200030, China; Co-innovation Center of Neuroregeneration, Nantong University, Nantong, Jiangsu 226007, China; Department of Oncology, The First Affiliated Hospital of Anhui Medical University, Hefei, Anhui 230032, China

Fibroblast growth factor 21 (FGF21) is a hormone that can balance nutrient fluctuations, control metabolic processes, and maintain energy homeostasis. Endogenous FGF21 is produced by a variety of cell types, including hepatocytes, adipocytes, bone cells, myocardial cells, and pancreas cells, and act on various effector tissues such as brain, adipose tissue, liver, heart, and skeletal muscle.^1^ The target that FGF21 interacts with is the cell-surface receptor composed of FGF receptors in complex with the single-pass transmembrane protein β-Klotho. These receptors are abundantly expressed, both in peripheral tissues and some regions of the CNS, such as the hypothalamus and amygdala and the locus coeruleus (LC).^2^ Physiologically, FGF21 can be induced by various metabolic stresses, including hunger, protein deficiency, monosaccharides, and alcohol, by acting on β-Klotho receptor in corresponding tissues, ultimately exerting regulatory effects (Fig. 1).

**Figure 1:**
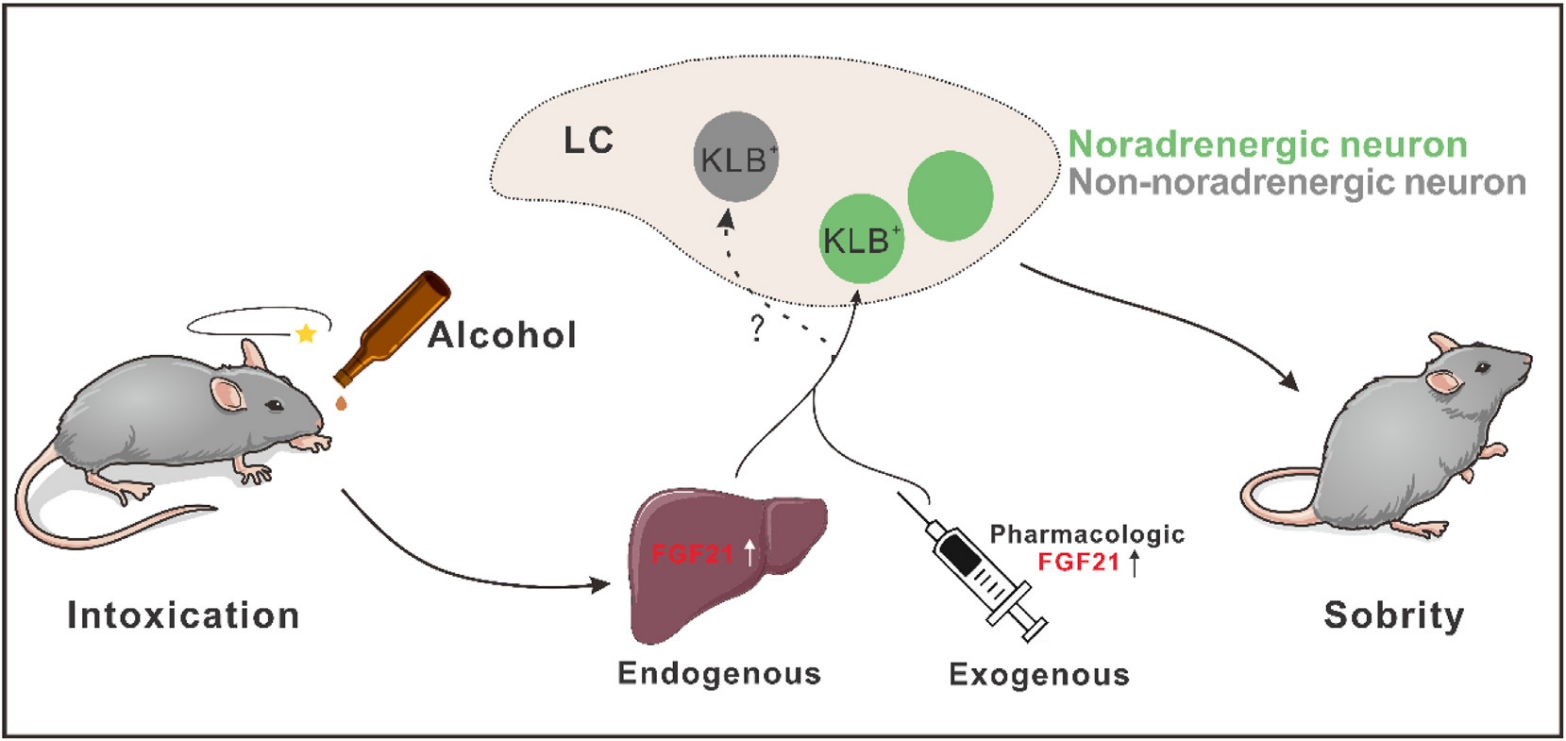
Schematic of FGF21 accelerating alcohol arousal with a liver-brain axis. Alcohol ingestion promotes liver secretion of FGF21. The increase of FGF21 in endogenous and exogenous manners will activate the noradrenergic KLB^+^ neurons in the locus coeruleus (LC). The activation of the norepinephrine system coordinates the mice's recovery from alcohol intoxication at last. However, whether the non-noradrenergic KLB^+^ neurons in LC have a role in anti-alcohol intoxication is still unknown.

Studies have shown that alcohol is by far the most effective trigger of FGF21 in humans. The elevated FGF21 is believed to be a protective response of the body to alcohol stimulation. Subsequent pharmacological experiments have confirmed that elevating FGF21 expression in the liver can reduce alcohol consumption and it is specially by the FGF21 signals to KLB-expressing neurons in the basolateral amygdala, which project to the nucleus accumbens.^3^ These studies emphasized the preventive and effective responses of FGF21 signaling from the liver to the brain to regulate the negative effects of alcohol. However, alcohol intoxication is the primary factor causing damage to the body during alcohol use, and whether FGF21 can mitigate alcohol intoxication remain unknown.

A recent study published in *Cell Metabolism* by Choi et al^4^ demonstrated that FGF21 regulates the recovery of mice from alcohol intoxication by activating noradrenergic neurons in the LC region. Using wild-type (WT) and global Fgf21^−/−^ mice, the authors first showed that the FGF21 deficiency exacerbated alcohol-induced intoxication, as Fgf21^−/−^ mice have a prolonged righting reflex recovery time after being administered a high dose of alcohol. Then, the author designed a comparative experiment to dissect the transmission characteristics of FGF21 in mice. Hepatocyte-specific Fgf21-knockout and neuron-specific KLB-knockout mice both exhibit longer righting reflex recovery times after being administered a high dose of alcohol. These results illustrate that liver-source FGF21 can facilitate the recovery from alcohol intoxication by acting on its receptor KLB in the nervous, which indicates a liver-brain axis underlying the effect of the FGF21 signal. Interestingly, pharmacologic FGF21 only accelerated the recovery from alcohol administration but did not affect ketamine, diazepam, and pentobarbital sedation.

Previous studies strongly confirmed that alcohol administration can significantly activate neurons in the LC region, which is the principal site of norepinephrine (NE). More importantly, deficiency of NE could prolong alcohol-induced righting reflex recovery time, similar to the result in Fgf21^−/−^ mice. Therefore, the authors further verified whether FGF21 can regulate alcohol arousal by acting on NE neurons in LC. Indeed, alcohol significantly activated cholinergic neurons in LC, and this phenomenon was not found in FGF21-deficient mice. By testing the co-expression of KLB-Tomato and norepinephrine transporter (NET), it is suggested that KLB is mainly expressed in the NE neurons, but not all. Similarly, pharmacologic FGF21 can also activate noradrenergic neurons in the LC, and in the neuron-specific KLB^Camk2a^ mice; there was no induction of c-Fos by FGF21. These results indicated that FGF21 can act on the NE neuron in LC directly.

Finally, by constructing transgenic mice Dbh^Camk2a^ (Dbh: dopamine b-hydroxylase, a raw material for synthesizing NE) and in KLB^Dbh^ (specific knockout KLB in the NE neuron), the authors found that these mice were able to abolish the acceleration of alcohol arousal induced by FGF21. Administration of the AAV-Cre virus into KLB^fl/fl^ mice's LC region could also eliminate the effect of FGF21 on the righting reflex. Notably, the selective α1-and β-adrenergic receptor antagonists prazosin and propranolol could block the intoxicant effect induced by FGF21. In summary, these results suggest that FGF21 can protect against ethanol-induced intoxication by the liver-brain pathway to act on noradrenergic neurons in the nervous system.

However, there are still several unclear questions that need to be investigated in the future based on this research. First, not all KLB-positive neurons are noradrenergic in LC, and they may also be the GABAergic or glutamatergic neurons. Interestingly, the GABAergic neurons in LC also play an important role in controlling arousal behavior.^5^ If the GABAergic KLB^+^ neurons in LC are the target of FGF21's signal is still unclear. In addition, the activities of NE neurons in the LC are regulated by the cortex, such as mPFC. Therefore, if there is a related circuit underly the FGF21 signal to LC noradrenergic neurons remains to be investigated. Last but not least, whether FGF21 is evolutionarily conservative in its response to alcohol. Studies have demonstrated that FGF21 can regulate alcohol behavior in mice, rats, and monkeys^3^; more importantly, the FGF21-KLB signal also is associated with alcohol consumption in humans. The anti-alcohol intoxicant induced by FGF21 may be translated to humans in the future.

Choi's study shed light on the mechanism of FGF21 signal anti-alcohol intoxication and how it acts on the nervous system from the peripheral liver. This study has strongly promoted the research on the physiological effects of FGF21 and also provided a new research approach for alcohol use disorders, especially the interaction between the peripheral and central nervous systems, which will be a focus of future research.

## Conflict of interests

The authors declare no conflict of interests.
